# Synthesizing Efficiency Tools in Radiotherapy to Increase Patient Flow: A Comprehensive Literature Review

**DOI:** 10.1177/11795549241303606

**Published:** 2024-12-13

**Authors:** Duvern Ramiah, Daniel Mmereki

**Affiliations:** Division of Radiation Oncology, Department of Radiation Sciences, School of Clinical Medicine, Faculty of Health Sciences, University of the Witwatersrand, Johannesburg, South Africa

**Keywords:** Radiotherapy, cancer treatment, efficiency tools, patient care, artificial intelligence, knowledge-based planning, scripting

## Abstract

The promise of novel technologies to increase access to radiotherapy in low- and middle-income countries (LMICs) is crucial, given that the cost of equipping new radiotherapy centres or upgrading existing machinery remains a major obstacle to expanding access to cancer treatment. The study aims to provide a thorough analysis overview of how technological advancement may revolutionize radiotherapy (RT) to improve level of care provided to cancer patients. A comprehensive literature review following some steps of systematic review (SLR) was performed using the Web of Science (WoS), PubMed, and Scopus databases. The study findings are classified into different technologies. Artificial intelligence (AI), knowledge-based planning, remote planning, radiotherapy, and scripting are all ways to increase patient flow across radiation oncology, including initial consultation, treatment planning, delivery, verification, and patient follow-up. This review found that these technologies improve delineation of organ at risks (OARs) and considerably reduce waiting times when compared with conventional treatment planning in RT. In this review, AI, knowledge-based planning, remote radiotherapy treatment planning, and scripting reduced waiting times and improved organ at-risk delineation compared with conventional RT treatment planning. A combination of these technologies may lower cancer patients’ risk of disease progression due to reduced workload, quality of therapy, and individualized treatment. Efficiency tools, such as the application of AI, knowledge-based planning, remote radiotherapy planning, and scripting, are urgently needed to reduce waiting times and improve OAR delineation accuracy in cancer treatment compared with traditional treatment planning methods. The study’s contribution is to present the potential of technological advancement to optimize RT planning process, thereby improving patient care and resource utilization. The study may be extended in the future to include digital integration and technology’s impact on patient safety, outcomes, and risk. Therefore, in radiotherapy, research on more efficient tools pioneers the development and implementation of high-precision radiotherapy for cancer patients.

## Introduction

**Figure 1.**
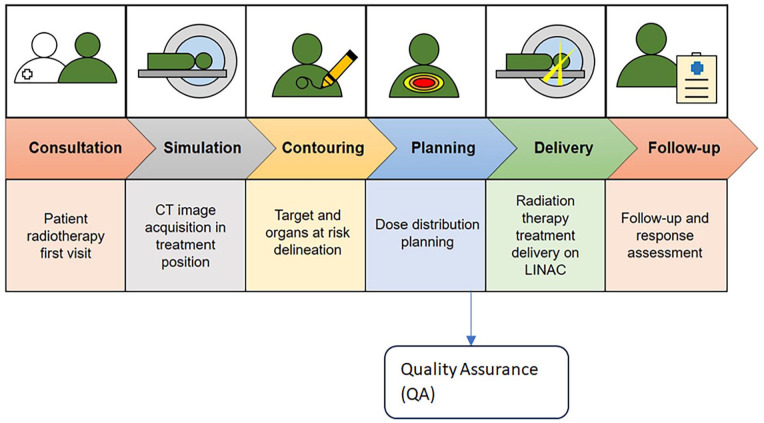
An illustrative representation of radiotherapy workflow. Source: Adapted from Marvaso et al.^8^ CT, computed tomography; LINAC, Linear Accelerator.

Radiotherapy (RT) is a crucial aspect of cancer treatment,^1^ with approximately 50% to 60% of cancer patients receiving it as part of their treatment.^2,3^ It necessitates significant financial resources, skilled professionals from various fields, high-precision equipment, and a specific organizational structure both externally and internally.^1^ It is indicated that the process of radiotherapy treatment consists of six fundamental stages: initial consultation, simulation, treatment planning, delivery, verification, and patient follow-up.^4^ It has been noted that the majority of indications for radiotherapy are associated with the treatment of cancer, and the implementation of a cancer control programme would be unattainable without the use of radiotherapy. In the last two decades, radiotherapy has rapidly advanced technologically, leading to improved precision in treatment planning and delivery.^1^ Studies have shown that RT is a cost-effective method for both definitively treating and palliating many cancers.^5-7^ An illustration of a comprehensive standard RT workflow is shown in Figure 1. It should be mentioned that in the typical RT workflow, the first step involves segmenting computed tomography (CT) image set. This is followed by positioning and optimizing radiotherapy beams, also known as treatment planning. It is important to note that automation typically follows a similar workflow, where each task is automated separately.^9^

The adoption of technology to optimize treatment planning across healthcare institutions has been improving the efficiency of the patient flow and overall planning process.^10^ This transformation has resulted in improved radiation oncology treatment planning workflow,^11^ improved safety in radiation oncology, shorter wait times for patients, and improved patient experiences.^12^ Additionally, it has also led to the reduction in the number of next-day plan quality assurance (QA) tasks^13^ and the ability to predict potential challenges and adapt plans based on individual patient characteristics.^12^ As a result, the RT workflow is designed to decrease workload,^13^ streamline planning processes, and enhance the capacity to handle a larger number of patients.^11,14^ However, healthcare facilities face challenges in efficiently managing the workflow of their patients during radiation therapy planning, highlighting the need for improvement.^15^ The nexus between the utilization of advanced technology and cancer care will address the gap in the RT workflow.^16^ Consequently, a new technological paradigm has evolved: artificial intelligence (AI)-driven tools. They aim to minimize delays in planning, improve patient flow efficiency through remote RT planning workflow, and extend the OARs delineation, target contouring, beam design, and RT plan optimization.^9,17^ In addition, new technology developments for RT workflow have led to the application of hybrid remote and AI-driven radiotherapy methods.^18^

The latest technological advancements in RT workflow provide the ability to address existing treatment planning challenges using real-time interactive planning. This technology has the capability to generate higher-quality plans in comparison to those generated by manual planners.^15^ There is a lack of comprehensive analysis in the current literature on technology-driven RT workflow.^19^ Over the last decade, several scholarly research investigations have resulted in technological advancements in RT workflow. This development has required a careful reassessment of various aspects of patient care, including initial consultation, simulation, treatment planning, delivery, verification, follow-up and other associated procedures.^19,20^ Previous literature reviews on RT workflow have mostly concentrated on strategic elements and have not adequately addressed the integration of automated planning process: advanced treatment planning software in RT workflow, resource scheduling and patient prioritization, and real-time monitoring and QA in treatment-planning processes. It may be argued that the previous research on RT workflow mostly focused on optimizing the methodologies, technologies, and challenges related to the RT planning process.^9,18^ Likewise, previous research has focused on fragmented investigations on AI-driven methodologies in RT workflow,^21,22^ knowledge-based,^23,24^ and scripting.^25,26^ Only studies conducted by Marvaso et al,^8^ Manson et al,^21^ and Munbodh^27^ specifically focused on comprehensive analyses of the advancements in augmented reality/virtual reality (AR/VR), technology-driven research in RT workflow, AI, and real-time analysis to enhance the RT planning process in clinical settings. Nevertheless, institutions all over the world have been primarily focused on utilizing technology as an effective strategy to enhance educational standards, improve the skills of health workers, promote patient well-being, conduct technology-driven research in RT workflow, and analyze compliance and scoping review analysis. Therefore, previous research has not provided a comprehensive synthesis of the technological advancements in RT planning and areas related to cancer research. With this study, the need of synthesizing the pre-published literature is addressed pertaining to the technological advancements in RT planning process. It aims to synthesize a comprehensive understanding of the challenges associated with technological advancements in the RT workflow.

Efficient tools in a radiotherapy department can significantly improve patient flow and enhance the delivery of high-quality and minimally invasive radiation treatments in low-resource settings. As such, efforts have been made to streamline the various steps of RT workflow that typically rely on in-person physicist expertise, physicians and dosimetrists: RT plan optimization, beam design, target contouring and isocenter marking, and target positioning.^28-30^ The lack of technological advancements, integration of digital systems, and implementation of real-time automation negatively affects cancer treatment and results in delays. This is crucial for improving patient throughput (volume) and optimizing the efficiency of systematic planning procedures (quality).^31^ Likewise, effectively addressing cancer in low-resource settings presents a significant challenge that requires the integration of technology. Health institutions frequently endeavour to partner with private technology organizations to implement technology-driven initiatives in patient flow in RT workflow. The accessibility of high-quality cancer care greatly relies on technological advancements in the RT workflow and information technology infrastructure.^31^ To effectively manage cancer, it is critical to analyze technological advancements in the patient flow and RT planning workflow. This study also aims to explore how some of the technological advancements in radiotherapy workflow and the automation of some of these processes could streamline the radiotherapy planning process, requiring fewer professionals to complete the process and, as a result, increasing access by expanding the number of places where radiotherapy is now available. Therefore, the aim of this investigation is to answer the following research question:

**Research question**: *What is the present state of the research on novel technologies in radiotherapy to increase patient workflow?*

The study’s contributions are as follows: The current published research on strategies and technologies in RT workflow lacks an in-depth analysis. This study aims to contribute to the existing literature by exploring the integration of automation tools in treatment planning processes, utilization of advanced treatment planning software, and the application of machine learning and AI in RT workflow in cancer care. Optimizing appointment schedules, ensuring the availability of necessary resources, and prioritizing patients based on clinical urgency in a radiotherapy planning workflow. The present study employs a structured content analysis method to identify various efficiency tools documented in the literature on RT planning workflow.^32^ Importantly, this study focuses on identifying and analyzing various efficiency tools in RT planning workflow. These tools include AI-based auto-segmentation techniques, remote radiotherapy planning, scripting tools (knowledge-based planning), and hybrid of remote planning and knowledge-based planning/ scripting. These tools have potential applications in different contexts within the public health sector of cancer care. By examining these tools, research in treatment planning can gain insights into their potential benefits and drawbacks, ultimately helping treating facilities and organizations make informed decisions regarding their implementation in RT planning workflow.

This study aims to thoroughly examine the impact of efficiency tools in a radiotherapy department, specifically in terms of improving patient flow within the planning department. The study starts by providing an overview of the patient flow within RT workflow. This is followed by a detailed explanation of the research methods employed, the findings obtained, and a thorough analysis of the literature review. Finally, the conclusions of the study.

### Contouring in radiation therapy planning process

Contouring is a critical aspect of treatment planning.^33^ In the early days of radiotherapy, people lacked sophisticated treatment planning software and CT imaging. Radiotherapy planning initially used surface markups and 2D simulations, but with the introduction of CT scanners and CT simulation, contouring of radiation targets and OAR became necessary. This increased the time required for radiation oncologists to be involved in the treatment planning workflow. Automating this process could allow oncologists to focus on tasks requiring specialist input, reducing their workload time.^19,34^

With the evolution of CT and advancements in radiation oncology, treatment planning has become more precise.^34^ However, more precise contouring and dose calculation are still being developed to improve treatment outcomes.^35,36^ As such, CT scans have made patient care easier for radiation oncologists, allowing them to transfer patients to contouring stations, which are highly effective drawing tools.^37,38^ Thus, the evolution of CT and advanced development in the field of radiation oncology, as well as treatment planning, is well pronounced in the treatment.^34,39^ Advances in technology enable precise contouring and dose calculation, with modern contouring stations combining multimodal imaging like MRI radiotherapy workflow and CT, and positron emission tomography-computed tomography (PET-CT) scans for targets and critical structures.^40,41^ The integration of AI technology like RadAI from Siemens in radiation oncology has revolutionized treatment planning and delivery. The application of deep learning contouring algorithms has shown promise in improving automatic delineation for head and neck OAR, addressing limitations of existing clinical algorithms like atlas-based contouring. This advancement enhances precision and accuracy in organ delineation, as highlighted by a study by Dijk et al.^42^

Furthermore, Rhee et al’s^43^ study illustrated on the development of an AI-based automatic contouring system for cervical cancer using convolutional neural networks. The results of the study highlighted the capability of AI-based auto-contouring systems in accurately delineating clinical treatment volumes and normal structures essential for various radiation treatment planning techniques. In a recent study by Zabel et al,^44^ the authors delve into the clinical evaluation of deep learning and atlas-based auto-contouring of bladder and rectum for prostate radiation therapy. The study emphasized the time-saving advantages of using auto-contouring software for initial contour generation. Furthermore, it highlighted the significance of quantifying workload changes during the editing phase. A study by McCarroll^29^ assessed the accuracy of automated contouring of normal structures for patients with head and neck (HNC). The results of the study indicated that automated contouring generated normal structures and accurate contours, requiring only minimal editing. This suggests that the continuous use of automated contouring in developing automated radiation treatment planning algorithms is a viable option for clinical applications.^29^

A recent study by Wong et al^45^ examined the use of deep learning-based auto-segmentation in radiotherapy planning structures. The findings showed a closer similarity between the contours generated by the deep learning algorithm and those manually created by expert radiation oncologists. Specifically, this similarity was observed for OAR and clinical target volumes in the central nervous system, head and neck, and prostate. It is evident that AI-based has the capability to achieve a level of accuracy that is comparable to that of manual expert manual contouring. As such, radiation oncologists can now check and adjust these contours rather than contour everything from scratch, reducing the time spent on these procedures from several hours to minutes, allowing them more time to focus on patient care or evaluate radiotherapy plans.

Additionally, the study conducted by Shen et al^46^ underscored the inherent benefit of auto-contouring the clinical target volume for prostate cancer radiotherapy, which is the reduction in the contouring time of radiation oncologists. This emphasizes the efficiency improvements that are linked to AI-based auto-contouring, which contribute to enhanced productivity and a streamlined workflow in radiation oncology practices. For instance, thanks to AI, DL-based methods have been utilized to evaluate the accuracy of contouring of OARs and targets in lung cancer patients,^47^ as well as salivary glands in head and neck cancer patients.^48^

### Patient flow through radiotherapy workflow

**Figure 2.**
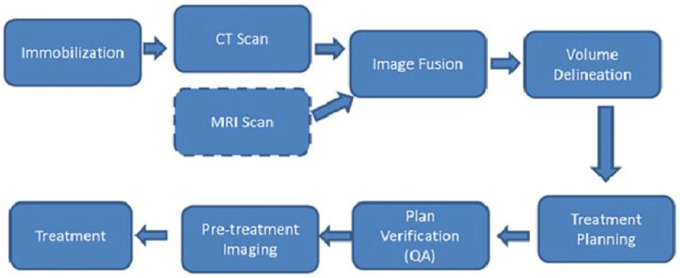
Radiation treatment planning and delivery workflow. Source: Adapted from Chandarana et al^53^ [PMC]. © 2018 International Society for Magnetic Resonance in Medicine. Licence number 5887760275626.

In order to ensure the continued provision of essential treatments, healthcare providers worldwide must make necessary adjustments to patient flows and restructure treatment pathways.^49^ Efficiently managing facility utilization and personnel workload throughout the treatment evaluation, planning, and irradiation processes can enhance appointment compliance and overall quality management.^49^ The patient flow in radiotherapy workflow depicts the process maps that encompass the entire duration, from the patient’s entry into the examination room to the completion of the check-out process.^50^ This could potential assist radiation oncologists in safely continuing and initiating therapy.^49^ As previously stated, the process of treatment planning workflow typically starts with obtaining a CT scan of patient upon admission for external beam radiation therapy (EBRT). In this case, from the CT, a dosimetrist or physicist can derive a highly conformal, patient-specific, three-dimensional (3D) treatment plan. The aim of this plan is to administer the physician’s prescribed dosage to the specified target area. It is indicated that the plan is created using sophisticated treatment planning software that accurately models the interaction between high-energy radiation beams and tissue. In addition, it computes the radiation dose administered to both the target area and surrounding tissues. The attending physician, medical physicists, and radiation therapists work together to ensure the accuracy, safety and effectiveness of the calculated treatment plan. As such, the treatment planning system transfers the plan to an electronic medical record system. The plan is then simulated and delivered to the patient using a linear accelerator (LINAC) in a radiation-shielded environment. This process is carried out by a team of radiation therapists, who may be under the supervision of a physician or medical physicist.^51^ Throughout the process, a team of experts from various subspecialties in radiation oncology collaborates to create and ensure the quality of the treatment plan, spanning from CT simulation to treatment delivery on the LINAC. Research has suggested that a clearly defined and manageable clinical workflow, effective communication among a patient’s care team,^52^ and coordinated care through real-time and automated resource tracking, work allocation, and completion all contribute to improving clinical efficiency and patient safety.^51^ Nevertheless, there are often significant time delays between the acquisition of CT scan and the actual treatment. To improve radiotherapy workflow, technological development solutions are broadly applicable in various settings with minimal disruption to existing operations. Figure 2 depicts the workflows involved in radiation treatment planning and delivery. It has been observed that modification or alterations may be necessary for certain disease sites when implementing these steps.^53^

## Methodology

This study conducts a comprehensive review of existing literature following some of the steps used in a systematic literature review (SLR), using content analysis, with a defined criterion, inclusion and exclusion criteria, and rationale to address the research questions. Content analysis is defined as ‘systematically collecting materials from major academic sources and sorting articles based on the scope of the review, and synthesising contents for emerging themes’.^54^ The analysis also identifies the existence of certain words, themes, or concepts within a given set of qualitative data, such as text. Indeed, content analysis is useful for quantifying and analyzing the presence of meanings. The present study examines the relationships between specific words, themes, or concepts within the radiotherapy workflow and the significance of efficiency tools in improving quality cancer care. An initial literature search was performed to identify previously published studies that examined the impact of efficiency tools in radiotherapy planning workflow. Table 1 shows the scope and delimitation of the study as suggested by Das.^32^ We conducted a comprehensive analysis of the selected publication to identify overarching thematic aspects. Subsequently, we categorized each publication into a specific theme. To ensure a systematic approach, we assigned articles based on the overarching thematic aspects of the study, even though there may have been some overlap in certain publications. After conducting a thorough analysis of each publication, several concepts and themes were identified in the efficiency tools used in the patient flow of the RT workflow process. Each tool of efficiency in the RT workflow process has several distinct advantages and disadvantages.

**Table 1.** Criteria for defining the scope and limitations.

Criteria	Inclusion	Exclusion	Rationale
1. No. of publication	Peer-reviewed articles	Non-peer-reviewed	The selection of high-quality research articles with academic rigour
2. Language	Publications in English	Publications not in English	Mostly read articles
3. Timeline	Till March 2024	Other than that	Efficiency tools to improve RT planning recently emerged, and the goal of this in-depth review is to provide the status of recent literature on the topic
4. Type	Journal articles	Blogs, magazines, news articles, and other grey literature	This This review justifies the inclusion of scholarly research articles of high-quality
5. Focus of the publication	The articles focus of the articles will be on issues related to efficiency tools to improve RT planning as the key objective	Articles refer to efficiency tools without referring to RT planning	The focus is to limit analysis to utilizing efficiency tools to improve RT planning

Source: Adapted from Das^32^ modified by the authors.

The search was conducted in the WoS, PubMed, and Scopus databases, widely recognized as significant sources of scientific literature, utilizing the ‘title, abstract, and keywords’ fields. The search query included the terms ‘efficiency tools’ AND ‘Artificial intelligence’ OR ‘remote radiotherapy planning’ AND ‘patient workflow in radiotherapy’ OR ‘knowledge-based radiotherapy planning workflow’ OR ‘scripting’ AND ‘radiotherapy workflow’ OR ‘RT’ OR ‘hybrid remote and knowledge-based/scripting’ (see Table 2 as suggested by Manson).^21^ During the initial search till February 2024, a total of 19,023 publications were retrieved from the Scopus, WoS and PubMed databases. Our selection criteria only included peer-reviewed journal articles published in the English language. We conducted a comprehensive review of 91 articles, primarily based on abstracts and introductions, and further analyzed the content of these publications.

**Table 2.** The summary of the literature search strategy.

Items	Specifications
Date of search	November 22, 2023, to March 18, 2024
Databases and other sources searched	Scopus, PubMed, Web of Science
Search terms and Boolean operators	‘Tools of efficiency’ AND ‘Artificial intelligence’ OR ‘remote radiotherapy planning’ AND ‘patient workflow in RT therapy’ OR ‘knowledge-based RT planning’ OR ‘scripting’ AND ‘radiotherapy’ OR ‘RT’ OR ‘hybrid remote and knowledge-based/scripting’
Publication timeline	Till February 2024
Inclusion and exclusion	The review included publications in English. Publications in other languages in languages other than English were not included
Selection process	The search was carried out by each author independently

## Results

**Figure 3.**
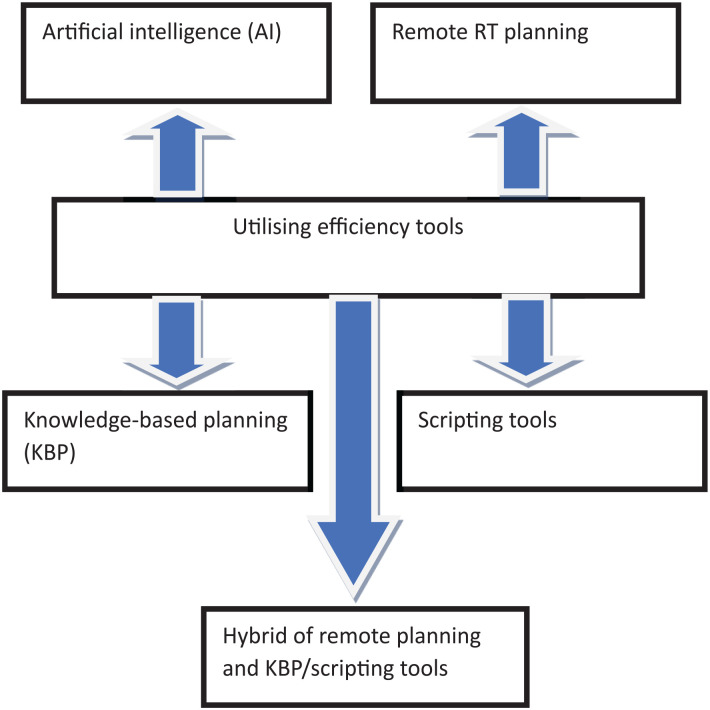
Efficiency tools for improving patient workflow. Source: Adapted from Mateko modified by the authors. Mateko.^63^ © 2024 The Authors. The Electronic Journal of Information Systems in Developing Countries published by John Wiley & Sons Ltd. License under [CC BY-NC-ND 4.0].

This section presents the findings of this study. Based on this study synthesis, various scholars have noted that AI-based auto-segmentation techniques,^19,55,56^ remote radiotherapy planning,^57^ scripting tools,^58^ knowledge-based planning,^59^ and hybrid of remote planning and knowledge-based planning/scripting tools^60^ are possible solutions to improve patient flow and reduce workload in RT workflow. Efficiency tools have been proven to be beneficial in high-income countries (HICs), improving efficiency in RT planning workflow in the health sector.^61,62^ In other words, efficiency tools play a crucial role in HICs in the process of RT workflow. As such, benefitting countries that apply them in cancer treatment and other fields and advancing technology. Figure 3 depicts the efficiency tools utilized in this study for improving patient flow in RT workflow. These tools are described in the preceding sub-section.

### Possible solutions

Technological advancements, notably generative AI technologies, have made it possible to automate and speed up the process of RT workflow, allowing the limited number of professionals to concentrate on specific tasks.^15^ Several studies (Chow et al^64^; Sivayogan et al^65^; Chow et al^66^) have analyzed the creation of computing tools such as chatbots, Internet of Things (IoT) apps, and graphical user interfaces (GUI) in radiotherapy, showing that these tools are designed to streamline and enhance the efficiency of various aspects of cancer treatment. Although the study by Chow et al^64^ found that chatbots address challenges such as data privacy, accuracy, and the integration of AI technologies into routine medical practices, highlighting both the benefits and limitations of these innovations in oncology care, further research is needed to explore this. Regarding the GUI for electron monitor unit calculators, two studies (Sivayogan et al^65^; Chow et al^66^) found that utilizing these tools significantly improves accuracy in dose calculations and precision in radiation dose planning, thus enhancing the effectiveness and precision of electron therapy treatment. This also simplifies the calculations for clinicians, ensuring a more efficient and reliable delivery of electron therapy in medical settings. Similarly, Pearse et al,^67^ in a study on ‘An Internet of Things App for Monitor Unit Calculation in Superficial and Orthovoltage Skin Therapy’, revealed that an IoT-based application can help clinicians improve the accuracy of monitor unit calculations for skin therapy, enhance treatment planning, and increase precision in radiation. This streamlines clinical workflows, reduces human error, and improves patient outcomes by providing reliable and efficient monitor unit calculations through connected devices.

Researchers have indicated that radiation oncology is an intricate field that requires the application of large language models to embark on a new era characterized by improved patient care and clinical efficiency,^68-71^ as well as informed recommendations tailored to the specific needs.^72^ Planners can now design highly complex plans with the aim of minimizing harm to normal tissue while still ensuring effective control of the tumour, thanks to the automation of radiotherapy workflow.^56^ Below is the discussion of current state of efficiency tools as part of the study’s empirical literature review.

### Artificial intelligence-based auto-segmentation techniques

**Figure 4.**
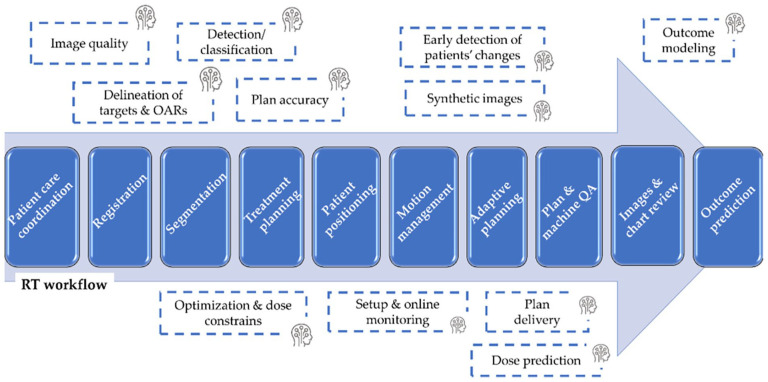
An illustration depicting the steps involved in the patient-based workflow for radiation therapy, highlighting the potential application of artificial intelligence (AI) techniques. Source: Adapted from Santoro et al^18^ Licensed under [CC BY].

Over the past few decades, AI have been utilized in various medical disciplines to address exceedingly complex and multifaceted challenges, including those in radiotherapy. The challenges in the workflow include tasks such as segmentation, the generation of synthetic images, and the prediction of treatment outcomes.^18^ Research has indicated that segmentation is a crucial component of image analysis, encompassing tasks such as detection, feature extraction, classification, and treatment.^73,74^ Tumour segmentation is the process of accurately identifying the spatial location of a tumour.^75^ The process of segmentation in radiotherapy workflow entails manual delineation of anatomical regions of interest, including tumour volumes and OAR, by clinicians.^36,76,77^ Although there are some drawbacks, such as the potential need for a significant amount of clinician time per patient and the possibility of variability between different observers, it is important to note that this method has been well documented. Computer algorithms are used in automatic segmentation to tackle these challenges.^36,78,79^ Auto-segmentation accelerates the treatment-preparation workflow by reducing the rate-limiting step^80-82^ and improving consistency in normal tissue segmentation.^80,83^ The development of these techniques aims to automatically segment OAR and target volumes, as well as radiation dose distributions. The automation aims to save, enhance consistency, and improve dose-volume parameters.^17,72^ AI-based techniques have the potential to significantly improve the RT patient-based workflow. The process is illustrated in Figure 4.

Table 3 provides a comprehensive overview of AI, including assisted, augmented, and autonomous types, and their characteristics in healthcare facilities across the human-machine intelligence continuum.^84-86^ Technologically, AI has the potential to improve the detection and diagnosis of malignant tumours by enhancing specificity, sensitivity, and diagnostic accuracy. Compared to traditional approaches, AI tools exhibit greater potential with their higher feasibility and accuracy when using various technologies, including computer-assisted detection (CAD) systems and deep learning algorithms.^87^ More detailed descriptions of the three types of AI, along with practical examples from real-world and medical contexts, can be found in previous studies.^84,87,88^ However, LMICs primarily rely on assisted AI. In terms of autonomous AI, this development has not yet taken place.

**Table 3.** The human-machine intelligence continuum.

Type of intelligence	Definition	Level of human involvement	Example	Example in healthcare
Assisted	System providing and automating repetitive tasks	Little or none	Industrial robots	UR robots for blood work (Copenhagen Hospital)
Augmented	Humans and machines collaboratively make decisions	Some or high	Business analytics	Watson for Oncology (Memorial Sloan Kettering)
Autonomous	Decisions made by adaptive intelligent systems autonomously	Little or none	Autonomous vehicle	IDx-DR for retinal images (University of Iowa)

Research has noted the use of AI-based tools in automating support for different aspects of treatment planning, radiotherapy delivery, and treatment response assessment.^89,90^ As such, the development of algorithms has been a significant focus, aiming to automate the planning process and/or optimize dosimetric trade-offs. These algorithms have significantly improved treatment planning efficiency and ensured the consistency of plan quality in radiotherapy workflow.^72^ AI-based treatment planning algorithms are reportedly aimed at improving treatment quality. In this scenario, research envisioned AI performing all human-performed operations and reasoning logics through a comprehensive analysis of the patient’s anatomy, seamlessly integrating parameters like prescription and delivery technique into its decision-making process.^56^ Consequently, various areas of medical imaging applications, such as tumour staging,^91^ image registration,^92,93^ automatic treatment planning,^94^ QA,^22^ outcome predictions,^95-98^ and others, have used AI-based auto-segmentation techniques.^72^ Research has suggested that ImageNet, a dataset of natural scenes, is a widely utilized for deep learning models in medical image investigations, particularly convolutional neural networks (CNN).^92^

Earlier studies have indicated that the precise segmentation of OARs and target volumes is essential for effective radiation treatment planning.^22^ Recent studies have indicated that incorporating CNNs into auto-segmentation models can improve the consistency and efficiency of RT workflow process.^99,100^ The auto-segmentation process classifies each voxel in an image as either part of an OAR or target, based on its position, intensity properties, and surrounding characteristics.^55,101,102^ Savenije et al,^103^ suggested that these models currently outperform conventional auto-contouring methods and are comparable to the accuracy achieved through manual delineations. For instance, a study explored the potential time-saving benefits of using software-generated contouring as a starting point for manual OAR contouring for lung cancer patients. The results showed that adjusting software-generated contours proved to be an effective strategy for reducing the contouring time of OARs for lung radiotherapy while conforming to local clinical standards.^100^ Based on research, AI-based segmentation has been found to decrease inter-observer variability in delineating OARs and targets. As a consequence, researchers have applied machine learning (ML) and deep learning (DL)-based algorithms to perform automatic image segmentation, demonstrating their ability to mimic the results achieved by expert radiation oncologists in accurately delineating and identifying OAR and tumours.^18^

It is noteworthy that AI-based auto-segmentation techniques in automatic treatment planning (ATP) are a significant advancement in the field of radiation therapy. These techniques primarily use DL-based methods with deep architecture and compositionality.^17,104^ The proposed change will streamline future RT workflow, reducing the need for human planners and physicians, thus improving human-centred clinic care tasks.^104^ These tools are developed using data obtained from thousands of patient scans, which have been meticulously trained to ensure accuracy. Research suggested that collaboration among radiation physicists, radiation oncologists, radiation dosimetrists, radiation therapists, and other personnel is crucial for developing realistic ATP workflow for AI training in radiotherapy plan generation and verification.^56^

Furthermore, research in medical imaging has significantly advanced image-based AI applications, a major area in ATP research. One particular area of focus is on enhancing the accuracy and efficiency of dose prediction by predicting dose distribution.^56^ However, it is important to note that most recent AI studies in radiation oncology primarily focused on predictions, with fewer studies exploring treatment planning reasoning simulations.^105^ In some instances, ATP sometimes employs generative adversarial networks, which are algorithms that generate representative samples from training data by implementing two networks that compete with a zero-sum task.^106^ Generative adversarial networks have successfully implemented medical image segmentation, disease diagnosis, and radiotherapy dose distribution.^107,108^

In a research study, the author explored the validation of 3D CNNs for semi-automated delineation of OARs in HNC. The authors found that automated delineation significantly reduced the average correction time by 33% compared to manual delineation (23 vs 34 minutes) (*P* < 10–6),^99^ indicating enhanced efficiency and consistency. AI-based auto-segmentation techniques offer advantages such as user preferences output adjustments, reduced human intervention, and improved plan quality and consistency.^109,110^ They also analyze treatment techniques, minimize bias, evaluate study eligibility, promoting shared/informed decision-making for personalized treatment planning (e.g. patient selection).^56,111^ However, it also presents several challenges. Smaller sample sizes pose a number of challenges, including a smaller sample space, difficulties in patient recruitment, variations in data acquisition, a lack of infrastructure, and the labour-intensive image processing required by human experts.^16,90^ The specific types of radiotherapy plans needed in ATP applications further amplify these challenges. Furthermore, simulating ATP workflow is highly demanding in terms of computational resources and requires validation prior to its integration into regular clinical practice.^56,72^ The limitations of AI-based auto-segmentation techniques include high costs, ethical and legal barriers,^18^ the need for advanced skills, and the fact that understanding imaging modalities is crucial for detecting artefacts and determining their underlying causes. This is also due to increased examination time or ethical guidelines; the decline of domain knowledge among physicians, physicists, or radiation therapists reduced their ability to gain experience in creating manual plans or segmentations in the clinical workflow, necessitating model change (change over time in terms of fractionation schemes, devices, etc).^22^ Understanding how models operate and interpreting their results is crucial,^68^ as is establishing complex agreements for exchange of data, scripts, and models across multiple centres, requiring a clear understanding of their operation.^112^

The application of ML and AI in radiotherapy was assessed by Xu et al,^113^ Rebelo et al,^114^ and Siddique and Chow,^115^ who reported notable improvements in increasing precision, reducing treatment times, and minimizing human errors in radiotherapy. Additionally, in terms of AI-based chatbots, Rebelo et al^114^ found that patients’ knowledge significantly improved when AI-assisted tools were integrated into clinical practice, highlighting accessible and reliable information as a key benefit. Another study by Xu et al^113^ found that chatbots for healthcare and oncology enhanced patient support, diagnosis, symptom management, and treatment guidance. Conversely, the study noted challenges related to data privacy and accuracy in chatbot use. Therefore, assessing the complications of these tools is vital for cancer treatment, as it directly impacts patient safety. However, the study emphasized the need for better integration of AI technology into healthcare systems while showcasing its potential benefits for oncology care.

It is suggested that one of the primary challenges is ethical consideration when implementing AI techniques and their expanding functions.^116^ For example, research indicates that human supervision is crucial for effectively managing and controlling the AI findings, especially in patients receiving RT, as AI continues to develop.^18^ As a result, protecting patient data privacy is crucial throughout the process, as transferring patients scans across borders may violate laws in some countries. Planners can avoid this by logging in remotely and not storing any patient data on their servers.

### Remote radiotherapy treatment planning

**Figure 5.**
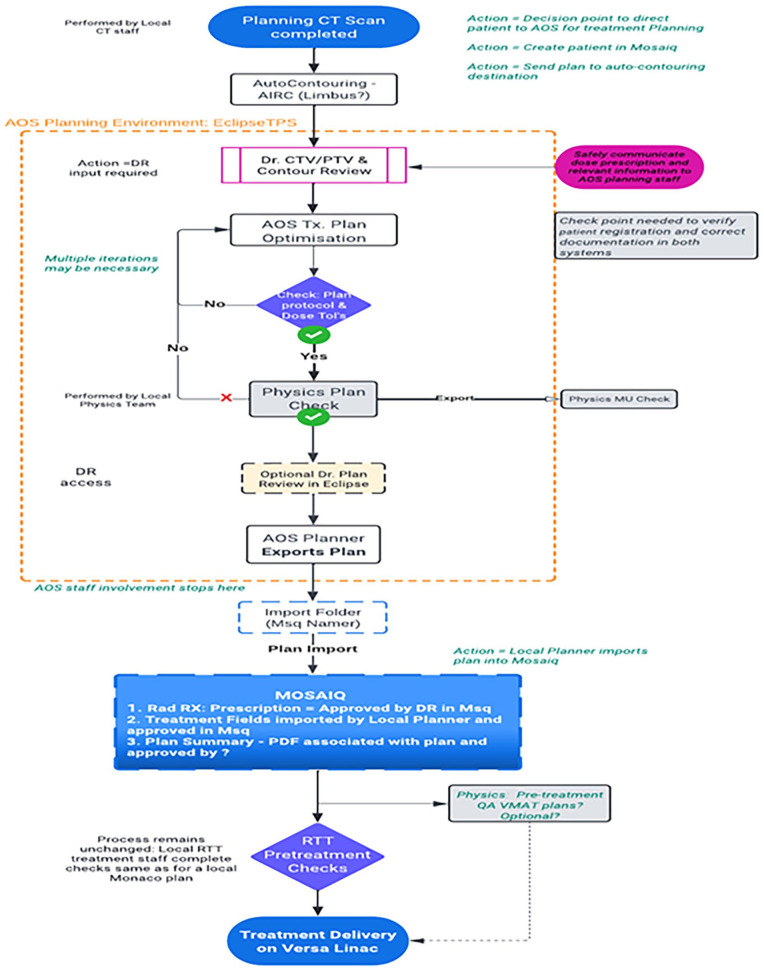
Steps of RT based on remote radiotherapy planning. Source: Constructed by authors from a summary of CMJAH and Siemens Varian Project by Ramiah et al^119^ (Siemens Varian).

The process of radiotherapy treatment planning is highly intricate, involving various factors such as patient factors, tumour characteristics, physician requests, contouring, software, and treatment planner experience. Collectively, these factors influence the final treatment plan. This emphasizes the importance of high-quality and seamless RT workflow.^117^ The development of remote radiotherapy applications like radiation planning assistant (RPA) has revolutionized RT treatment plans. RPA is a web-based AI-assisted tool that provides fully automated creation of RT treatment plans. Traditionally, these steps required the expertise of an in-person physicist, along with the involvement of physicians and dosimetrists. The primary goal of RPA is to improve the accessibility of high-quality RT treatment planning system.^31^ Figure 4 illustrates another example of remote radiotherapy treatment planning system, which allows the generation of patients’ radiotherapy treatment plan at a site far from the location where the patient is scanned, and the treatment delivered. Studies have been carried out on the application of a fully automated creation of radiotherapy treatment plans, including volume-modulated arc therapy (VMAT) plans, for cancer patients.^29,30,86-90^ RPA has been shown to be technically effective in optimizing and generating radiotherapy treatment plans for cervical, head and neck, and breast cancer radiation therapy.^28-31^ A study found that RPA can generate clinically acceptable autoplans for head and neck (HNC) patients, potentially reducing the resources needed for RT treatment planning (particularly in LMICs, there is a significant shortage of radiation oncologists, physicists, and other necessary professionals to develop these intricate plans). The use of remote radiotherapy treatment planning implies that clinics with limited resources can more readily offer advanced procedures to their patients.^118^ The radiotherapy workflow in HNC involves the treating physician automatically delineating target structures on the contouring of the gross tumour volume (GTV). Additionally, the radiation oncologist must also review/edit automatically generated contours, make necessary adjustments where necessary, and delineate GTV.^117^ Another study evaluating the efficacy of a fully automated RT treatment planning (i.e., RPA) system for standard radiotherapy in treating cervical cancer has found it to be a highly effective technique. This study also suggested that the RPA system could provide a reliable option and improve access to high-quality RT in resource-constrained clinics (Figure 5).^28^

A study conducted by Santhanam^57^ explored a real-time remote multi-3D camera-based imaging system. The study demonstrated the potential of real-time patient setup visualization using stereoscopic imaging, enabling the expansion of high-quality radiation therapy in challenging environments lacking specialized expertise.^57^ Implementing an emergent after-hours radiation treatment process using remote treatment planning on optimal diagnostic CT scans has demonstrated the potential to enhance quality and safety by more accurately approximating the standard process of care.^120^ A study conducted by Cheung^121^ explored the advantages of intensity-modulated radiation therapy (IMRT), treatment delivery methods, and available equipment, while also discussing their limitations and ongoing development efforts to enhance the efficiency of the equipment and treatment techniques and procedures. The advantages of remote radiotherapy treatment planning include improved workflow, facilitating the transition from 2D to 3D treatments, transitioning from 3D to intensity-modulated radiotherapy (IMRT) treatments, reducing workload, shortening planning time, and expanding cancer treatment options.^121^ Pirzkall et al’s^122^ study compared conformal and intensity-modulated radiotherapy (IMRT) plans for nine patients. They found that IMRT techniques increase dose and target coverage while sparing OAR and can be delivered in a time frame comparable to other sophisticated techniques. The implementation of these solutions also has the potential to address staff and educational/training shortages, improve patient throughput (volume), and optimize the system planning process (quality).^31^ Limited investment from the staff and the availability of normal tissue autocontours can expedite the manual treatment planning process. This could ameliorate the expected staff shortages caused by a rising number of cancer cases in LMICs and improve access to care through the efficient creation of and completion of radiation radiotherapy treatment plans more rapidly. This could also give in-person employees more time to focus on other aspects of their work. These factors could be important in QA and clinical trial evaluation.^117^ It has been projected that the number of cancer cases will rise to 24.6 million by 2030.^123^ Previous research has indicated that remote radiotherapy treatment planning systems and telehealth could enhance accessibility to high-quality radiation therapy in areas with limited resources.^105,124^ The limitations of remote radiotherapy treatment planning systems include job stability, availability of internet connectivity, trust in an automated system, and appropriate disease paradigms.^31^ Another important consideration is that the planning system should be agnostic when it comes to RT treatment planning for different linear accelerators. Different units in LMICs use a variety of linear accelerators, and the system used should be compatible with all of them.

In summary, utilizing these technologies allows patients to have scans done at a site; auto-segment OARs; and radiotherapy planners can log remotely using the treatment site’s planning systems. Planners might be based anywhere, especially in countries with an abundance of professionals to undertake such RT treatment planning. Understanding the increasing complexity of RT treatment planning process is critical for reducing workload, delays, and planning considerations in treatment facility settings.

### Scripting tools (knowledge-based planning)

Previous studies have demonstrated the need to tailor RT treatment planning automation tools to meet the requirements of the practice. Numerous commercial treatment planning systems (TPS) enable the integration of user-specific automation into the RT planning workflow.^125^ Automatic RT treatment planning often employs script-based solutions.^126^ In scripting tools (knowledge-based planning), researchers predict dose in radiotherapy treatment planning.^127^ The integration of scripting into RT treatment planning systems has been proven to improve efficiency in routine tasks and has the potential to be utilized in clinical research. However, it is indicated that systems with a built-in scripting interface are the only ones that can use scripting.^128^ For example, commercial RT TPS like Monaco 6.0 (Elekta AB) provide scripting as a feature to users. The feature enables the user to create scripts to automate specific RT treatment planning activities^61^ using application programming interfaces (APIs), enabling them on multiple patients or treatment plans.^129^ Within Monaco, the user has access to scripting APIs that include Digital Imaging and Communications in Medicine (DICOM) import and DICOM export, contouring activities such as auto-margin and adapt anatomy, planning activities (such as creating a new plan from a template, adjusting beam setups, setting and adjusting plan objectives and constraints, setting and adjusting clinical objectives, performing calculation and optimization), and extracting plan data such as dose volume histograms (DVHs), statistics, reports, and QA plans.^130^

The Monaco 6.0 RT treatment planning system, Elekta, developed in Stockholm, Sweden, is extensively used in radiotherapy centres across the globe. It integrates Monte Carlo dose calculations with advance optimization tools to generate high-quality RT treatment plans for various RT techniques, including three-3D CRT, IMRT, VMAT, SRS, and SBRT.^129^ Huang et al^130^ developed an auto-planning platform to interface with Monaco and tested with VMAT planning for prostate and for head and cases. The results of the study showed that the improved efficiency and consistency of the plan quality can be linked to the clinical implementation of the auto-planning platform. Another study by Kodama et al,^131^ developed an automatic RT treatment planning system for the Monaco treatment planning system (Elekta AB, Stockholm, Sweden). The study aimed to optimize the RT treatment process for prostate cancer using VMAT, specifically by utilizing a single arc per treatment. The results of the study showed improved efficiency in managing the RT treatment planning system, optimizing human resources, and ensuring high-quality outputs. Monaco significantly outperformed the Pinnacle in terms of dosage distributions, therapy delivery parameters, and quality control outcomes, thanks to its versatile scripting functionality.^132^ The use of its scripting capabilities has also been reported to enhance the accuracy of dose calculation in RT TPS.^133^ The system also automates the generation and comparison of plans for both intensity-modulated radiotherapy (IMRT) and volume-modulated radiotherapy.^111^ Some TPS, including Eclipse (Varian Medical Systems, Palo Alto, USA), Pinnacle (Philips Healthcare, Eindhoven, Netherlands), and RayStation (RaySearch Laboratories, Stockholm, Sweden), have been developed and distributed to aid automated procedures, assisting physicians in the process. These systems, equipped with built-in scripting capabilities, aim to streamline tasks in the field.^25^

The Elekta Scripting API enables Monaco users to automate tasks within the RT treatment planning workflow. Thus, at best, scripting could potentially assist users in establishing and standardizing RT workflows in radiation oncology departments.^125^ For instance, breast radiotherapy treatment planning techniques use scripting in Raystation,^60^ demonstrating significant time-saving potential. As such, most tasks can be automated, which can improve RT workflow efficiency by reducing staffing. Funderud et al,^134^ found that clinicians predominantly preferred script-based automated radiotherapy treatment planning for cervical cancer because it provided acceptable target coverage, lower doses for most OARs, and more conformal dose distributions. This insight demonstrated how important scripting is in RT treatment delivery and how RT treatment planning could be harmonized, which is necessary for accurate radiotherapy, especially in resource-constrained settings. However, the scripting tools have drawbacks in that the user is still responsible for the final evaluation and interpretation of tasks performed by the scripting feature.^25^

In a nutshell, these tools are designed to automate certain aspects in radiotherapy treatment planning using predetermined algorithms. Much of the planning process for patients with specific types of cancers requiring treatment in the same area is generally consistent. Although these processes are the same for every patient, it does require a large amount of time for radiotherapy planners to perform their tasks.

### Hybrid of remote planning and knowledge-based planning/scripting tools

The hybrid of remote planning and knowledge-based planning/ scripting tools is a combination that automates most RT treatment planning processes, allowing remote planners to log onto the server to complete RT treatment plans/tasks that require human planners, allowing for more efficient and effective RT treatment planning. The efficiency of RT treatment planning depends on the significantly shorter time required to complete an RT treatment plan, which typically results is significantly reduced staff requirements. Here, the number of iterative plan adjustments needed to achieve a satisfactory treatment plan could be potentially reduced.^56^ Studies provided an in-depth overview of knowledge-based planning (KBP) and elaborated how predicting treatment plans or doses for new patients using past knowledge from previously optimized plans can lead to more efficient RT treatment planning. In literature, analyzing DVHs for a contoured structure or complete dose distributions can estimate the dosage.^135,136^

In a study by Peng,^126^ a commercially available hybrid planning solution was evaluated and compared with manual planning and script-based planning for rectal cancer patients. The authors found that hybrid planning, which integrates all parameters, when compared to manual planning, significantly reduced the dosage to OAR. The study suggested that hybrid planning offers better and more robust RT treatment plan quality compared to script-based planning.^126^ In research exploring the efficacy of hybrid automation in RT treatment planning (HAP) solution, which integrates KBP with script-based planning, it was found that HAP is highly effective in creating high-quality RT treatment plans for oesophageal cancer patients.^137^ A study was conducted to validate RapidPlan PT models for locally advanced head and neck cancer. The study utilized scripting to automatically generate proton and photon KBPs for 72 patients with recent oropharynx cancer. Results from the study showed that this approach can significantly expedite the RT treatment planning and patient-selection process while maintaining high quality of RT treatment plans.^58^ These findings were corroborated by the conclusions of Ling et al,^137^ Fan et al,^138^ and Xia et al^139^ (for lung cancer plans), who found that hybrid planning can improve robustness, quality and consistency of RT treatment plans compared to manual RT treatment planning. This suggests that hybrid planning has promise as a future approach to high-quality RT treatment planning for lung cancer. This is congruent with conclusion proposed by Momin et al,^23^ who suggested that the utilization of these tools enhances both the quality and efficiency of RT treatment planning compared to manually optimized RT treatment plans.

In conclusion, the RT TPS discussed above are hypothesized to reduce waiting time, workload, plan adjustments, and the number of professionals involved in the radiotherapy process. However, several questions emerged: is this true, and how much time will it save? Will the efficiencies gain at this stage of the RT treatment planning process result in bottlenecks in the RT treatment process?

To address these questions, a RT treatment planning system has been implemented at Charlotte Maxeke Johannesburg Academic Hospital, South Africa. Researchers propose a hybrid RT treatment planning solution that integrates remote and AI radiotherapy TPS for creating effective RT treatment plans. The investigation of RT treatment planning parameters to compare hybrid and manual RT treatment planning is ongoing. This, therefore, demonstrates the importance of evaluating the efficacy of remote RT planning in LMICs, notably in South Africa, in reducing time delays.

The integration of these technologies within the oncology community could offer promising solutions for delivering quality cancer care. For instance, the editorial titled ‘Advanced treatment planning strategies to enhance quality and efficiency of radiotherapy’ suggests that the automation of RT treatment planning can potentially achieve better RT treatment plan quality (on average) compared to manual RT treatment planners. The authors of the editorial suggested that clinics may struggle to provide high-quality, individualized treatment plans with manual RT treatment planning due to high patient loads and limited resources. The analysis indicated that automation of RT treatment planning is significantly driven by the potential for increased productivity.^15^ This demonstrated that the implementation of these efficiency tools in RT treatment planning (higher quality) could potentially reduce delays. This could lead to a reduction in workload, harmonization and standardization, ultimately reducing unwanted interpatient and inter-institution quality variations.^15^

## Discussion

The study provides a comprehensive analysis of significant advancement in the automation of RT treatment planning, including the use of efficiency tools such as AI, remote RT treatment planning, scripting tools, and a hybrid of remote planning and KBP. The implementation of efficiency tools is currently enhancing patient flow and reducing waiting times and workloads. As shown in most publications, the automation of RT treatment planning using efficiency tools generates safe, effective, and personalized treatments, enhancing patient care, potentially reducing disease progression risk, and enhancing efficiency and clinical benefits.

The study, utilizing PubMed, WoS, and Scopus databases, found that AI-based auto-segmentation tools, remote radiotherapy planning, scripting tools, and a hybrid of remote planning and KBP improved the efficiency of RT workflow, RT treatment plans, and quality cancer treatment. Studies indicate that artificial intelligence-based auto-segmentation techniques outperform manual planning in radiotherapy treatment planning for various cancer treatments,^56,72,89-93^ and delineation for OARs, whereas a study by Latif^69^ emphasized the importance of patient privacy. Studies on remote radiotherapy treatment planning have shown enhanced accuracy, precision, and treatment quality in various cancers, including head and neck,^118^ advanced cervical cancer,^28^ and prostate cancer when compared to manual planning.^132^ In the studies on knowledge-based RT treatment planning, the efficacy of the tool was provided. These crucial aspects include no iterations RT plan adjustments and reviewing/editing of plans, reduced workloads, and individualized RT treatment plans. With regards to scripting tools, like KBP, most publications show that they improve planning speed, increase consistency of plan quality, and reduce reliance on planner expertise. Previous studies suggested that a combination of remote planning and KBP/scripting tools can significantly enhance the RT treatment planning process. Ling et al,^137^ Fan et al,^138^ and Xia et al^139^ found that hybrid remote planning and KBP significantly improved RT treatment plan quality in oesophageal cancer patients, while Momin et al^23^ found combining these tools improved plan quality and efficiency in lung cancer compared to manually RT treatment plans. However, the findings may be inconclusive due to the limited research on these techniques in the RT treatment planning process.

This study provided significant theoretical and practical contributions to the literature. This study synthesizes literature on efficiency tools in radiotherapy treatment planning, especially AI remote radiotherapy planning, scripting tools, and a hybrid of remote planning and KBP. The study highlights the significance of technological tools in improving radiotherapy treatment delivery and digital transformation for efficient cancer management, enhancing patient workflow and expanding OARs. As such, literature analysis can provide practitioners with a comprehensive understanding of the key advancements in RT TPS.

Finally, automation of RT treatment planning enhances precision, accuracy, and safety and effective radiotherapy treatment plans. However, the literature on efficiency tools in cancer care is limited in scope. Future research should explore the automation of RT treatment planning, evaluate its effectiveness, and identify barriers and success factors. Additionally, exploring advancements and automation RT treatment planning in low-resource settings could broaden its scope.

### Limitations and strengths of the study

The current study has several limitations. First, this study identified a relatively small number of articles selected for review. However, the authors had to ensure that studies reviewed contained information about the automation of RT treatment planning.

Second, most of studies were on AI augmented techniques, and automation of RT treatment planning in cancers such as head and neck, breast, oesophageal, cervical. Because most of the articles focused on AI augmented techniques and the afore-mentioned cancers, which is only part of the application of efficiency tools in creation of RT treatment plans, little is known about contributions to waiting times taken to get treatment. The study’s limited scope may be attributed to the language criterion and the search strategy used. However, it could also reflect that the automation of RT treatment planning is not widely utilized in the healthcare facilities. In contrast, the number of studies on AI augmentation techniques in the automation of RT radiation planning was relatively large, which is attributed to its significance in the health sector, particularly ML tools. This highlights the urgent need to increase autonomous AI in RT TPS, ensuring the entire RT workflow is fully automated.

Third, studies included in this review provide valuable insights into the automation of RT treatment planning in healthcare facilities, but due to the limited global representation, these results cannot generalize across geographic regions or economic contexts. For instance, most of the investigations were primarily conducted in HICs. Only a few studies in low- and middle-income countries (LMICs) suggest the need for more research into the automation of RT treatment planning in resource-constrained countries. Although the application of AI, KBP and Scripting using Monaco 6.0 in RT treatment planning is primarily in HICs (insert studies from Sweden, Europe, and the United States), other regions may also use these efficiency tools in their healthcare facilities. Due to different healthcare settings, variations in the use of advanced technology, and differences in socio-economic context, legal, training and expertise, thorough empirical investigations are needed in different geographical and socio-economic contexts and among different organizational sizes and structures.

Fourthly, the meta-analysis was not conducted due to logistic constraints, but it is crucial for developing hypotheses based on prior results. It could have established a correlation between automation of RT treatment planning and cancer burden. Meta-analysis can limit or overcome biases of narrative reviews,^140^ but expertise-based subjectivity can also be a strength in systematic reviews and meta-analyses.

Last but not the least, the study emphasizes the significance of automating RT treatment planning and generating a comprehensive treatment plan. Therefore, the impact of automation in radiotherapy treatment planning (e.g. hybrid of AI and remote RT treatment planning) on reducing time delays requires further exploration.

Nevertheless, this study effectively synthesizes findings from various studies on the use of efficiency tools in RT treatment planning to enhance patient flow. This study offers a foundation for future research in this field, serving as a reference for researchers, healthcare managers, healthcare professionals, and policymakers.

## Conclusion

Technological advancements have had a significant impact on cancer care, highlighting the importance of efficiency tools in radiotherapy treatment planning for enhancing patient flow, reducing waiting times, reducing iterations in reviewing/editing plans, and addressing the growing cancer burden. These approaches have the potential to enhance the quality and efficiency of individualized, high-quality treatments. Collaboration between international organizations like the International Atomic Energy Agency, health ministries, and radiotherapy institutions is crucial. The study also highlights the potential benefits of integrating AI and automation in target contouring, beam design, and RT optimization, can minimize delays and improve QA. As such, systems such as Elekta Linacs with the Varian Eclipse treatment planning system can streamline the process, reducing delays and workload during treatment evaluation, planning, and irradiation.
